# Pulmonary Tuberculosis With Saddle Pulmonary Embolism and Deep Vein Thrombosis: A Rare Case Report

**DOI:** 10.7759/cureus.15958

**Published:** 2021-06-27

**Authors:** Eihab A Subahi, Mouhammad J Alawad, Elabbass A Abdelmahmuod, Dalal Sibira, Ijaz Kamal

**Affiliations:** 1 Internal Medicine Department, Hamad Medical Corporation, Doha, QAT; 2 Radiology Department, Hamad Medical Corporation, Doha, QAT

**Keywords:** pulmonary tuberculosis, pulmonary embolism, deep vein thrombosis, anti-tubercular therapy, anti-coagulation

## Abstract

Pulmonary tuberculosis is a common endemic disease in developing countries but its thrombogenic tendency is not well-studied and established yet. Pulmonary embolism is rarely reported in Mycobacterium tuberculosis infection. There are reports stating the relation of pulmonary embolism (PE) and deep vein thrombosis (DVT) with a severe infection of tuberculosis but no data is available to establish a mutual association between pulmonary tuberculosis and pulmonary thromboembolism. Herein, we report the case of a 51-year-old male who presented with a one-month history of productive cough, shortness of breath, and fever associated with chills and night sweating. He reported an 8 kg weight loss in the last month. He was found to have pulmonary tuberculosis. On further investigations for leg swelling and tachycardia. Deep vein thrombosis and sub-massive saddle bilateral pulmonary embolism were diagnosed, which was treated with thrombolysis therapy (alteplase). He responded well to initial therapy and was discharged on anticoagulation with anti-tuberculous therapy (ATT).

## Introduction

Tuberculosis (TB) is defined as an infectious disease caused by Mycobacterium tuberculosis [1]. TB can cause various symptoms and signs depending on the infected organs [1]. The lungs are involved in approximately 90% of cases while other systems can also be involved like the gastrointestinal tract, genitourinary tract, lymph nodes, bone, muscle, and central nervous system, and this is called extrapulmonary tuberculosis. In addition, it is well-known that a hypercoagulable state associated with tuberculosis may provoke thromboembolism, and this complication occurs in 0.6-1.0 of patients with tuberculosis [1-2]. Although venous thromboembolism (VTE) is considered a rare incident, it should be taken into consideration especially in those with severe pulmonary or disseminated tuberculosis, as there is some evidence that the risk of developing VTE increasing with the severity of the disease [3-4]. Another challenge is the drug interactions particularly between rifampicin, which is the mainstay of treatment, and warfarin or oral anticoagulants [5]. However, to the best of our knowledge, few cases have been reported in the literature about venous thromboembolism in patients with tuberculosis [1,3-4,6]. Therefore, physicians should have a high index of suspicion and PE should be suspected as one of the differential diagnoses in patients with pulmonary TB (PTB) who have hypoxia, tachycardia, sudden onset of chest pain, or increasing shortness of breath. As VTE is associated with high mortality and morbidity [4], therefore, it is important to perform an early diagnosis and initiate immediate and proper treatment [3].

## Case presentation

We present a case of a 51-year-old Bangladeshi male with a past medical history of uncontrolled diabetes mellites (DM) (last glycated hemoglobin (HBA1c) 13.4%) who was on oral hypoglycemic medication (metformin/sitagliptin). He came to the emergency department with a one-month history of productive cough, shortness of breath, which was associated with fever and night sweating. He reported an 8 kg weight loss in the last month. He denied any other complaints like hemoptysis. He works as a private house driver. He denied any contact with a patient with PTB.

On examination, he was spiking high-grade fever with a temperature of 39.2°C; tachypneic with a respiratory rate of 30 and tachycardic with a heart rate ranging between 120-130. His blood pressure was within normal limits. The patient was cachectic. Neck examination was unremarkable. Chest examinations showed decreased air entry in the left side in all lung zone with coarse crackles and dull percussion notes. The rest of the physical examination was unremarkable.

His laboratory findings on admission showed mild leukocytosis and anemia with white blood cells (WBCs) level of 11.8 x10^3/uL and Hb level of 11.4 mg/dL. platelet count with normal range. Other labs showed moderate hyponatremia with sodium level 124 mmol/L with CRP of high with value 140 mg/L. Renal and liver function tests were unremarkable.

**Figure 1:**
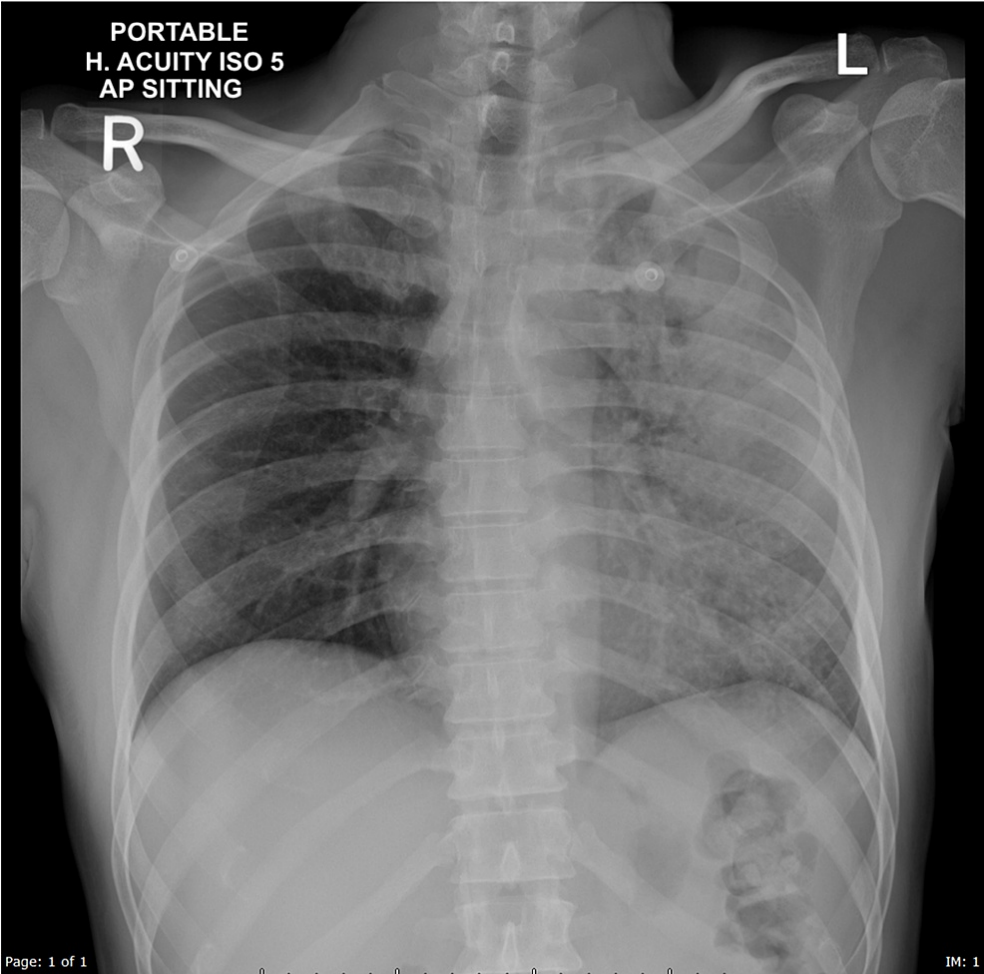
Frontal radiograph of the chest, demonstrating diffuse left lung field consolidation with an air bronchogram and evidence of a cavitary lesion in the left upper zone

Chest radiography showed evidence of heterogeneous opacity involving the whole left lung suggestive of consolidation and evidence of a cavitary lesion in the left upper zone (Figure 1).

His sputum smear for acid-fast bacilli (AFB) and polymerase chain reaction (PCR) was positive for Mycobacterium tuberculosis and was started on anti-tuberculous therapy (ATT). His HIV test was negative.

**Figure 2:**
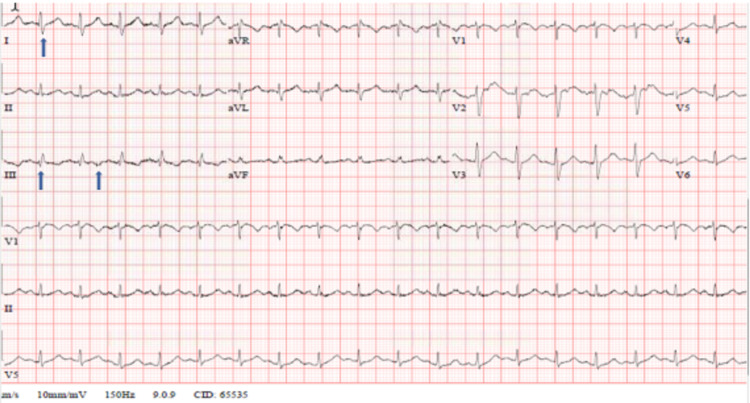
ECG strip showing the pattern of S1Q3T3 ECG: electrocardiogram

After 24 hours, the patient was still complaining of shortness of breath. Further assessment showed that he still had tachycardia. The further blood test showed raised troponin-T and pro-B-type natriuretic peptide (pro-BNP) with values of 24 ng/L and 5,634 pg/mL, respectively. The thyroid function test was normal. His electrocardiogram (ECG) showed sinus tachycardia with S1Q3T3 (Figure 2).

**Figure 3:**
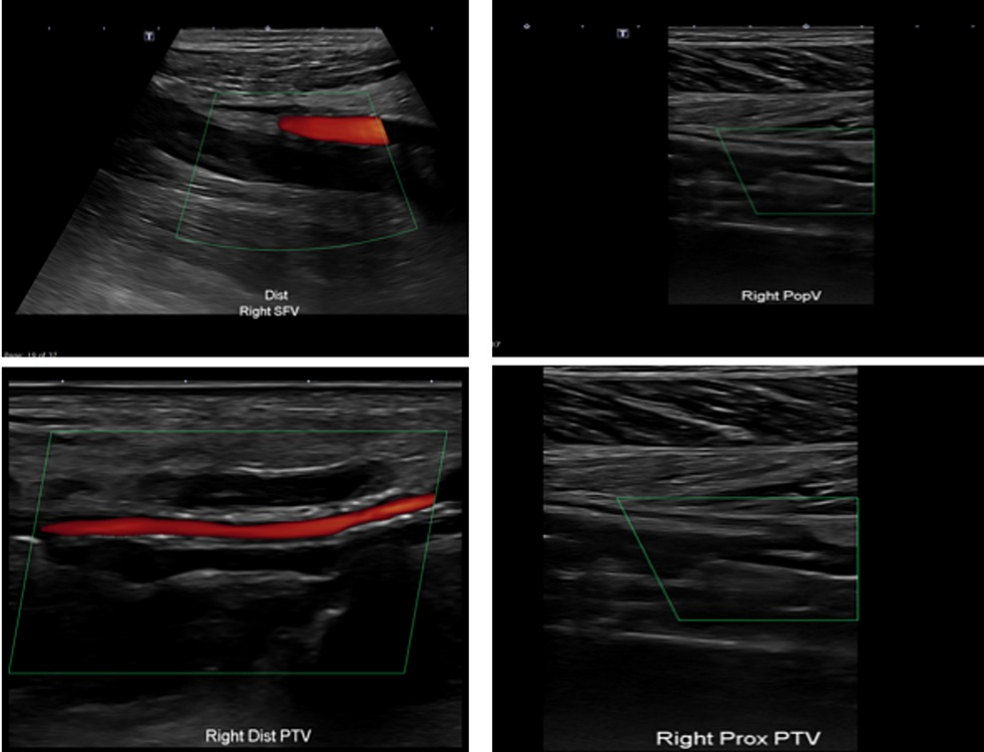
Right lower limb Doppler US scan showing no flow within the SFV, popliteal vein, proximal and distal PTV, compatible with right lower limb DVT SFV: superficial femoral vein; posterior tibial vein: PTV; DVT: deep vein thrombosis

Repeated physical examination revealed new mild right leg swelling, which was not present on the initial assessment. His Doppler ultrasound showed evidence of acute DVT from the distal posterior tibial vein (PTV) up to proximal superficial femoral vein (SFV) with areas of partial and no flow (Figure 3).

**Figure 4:**
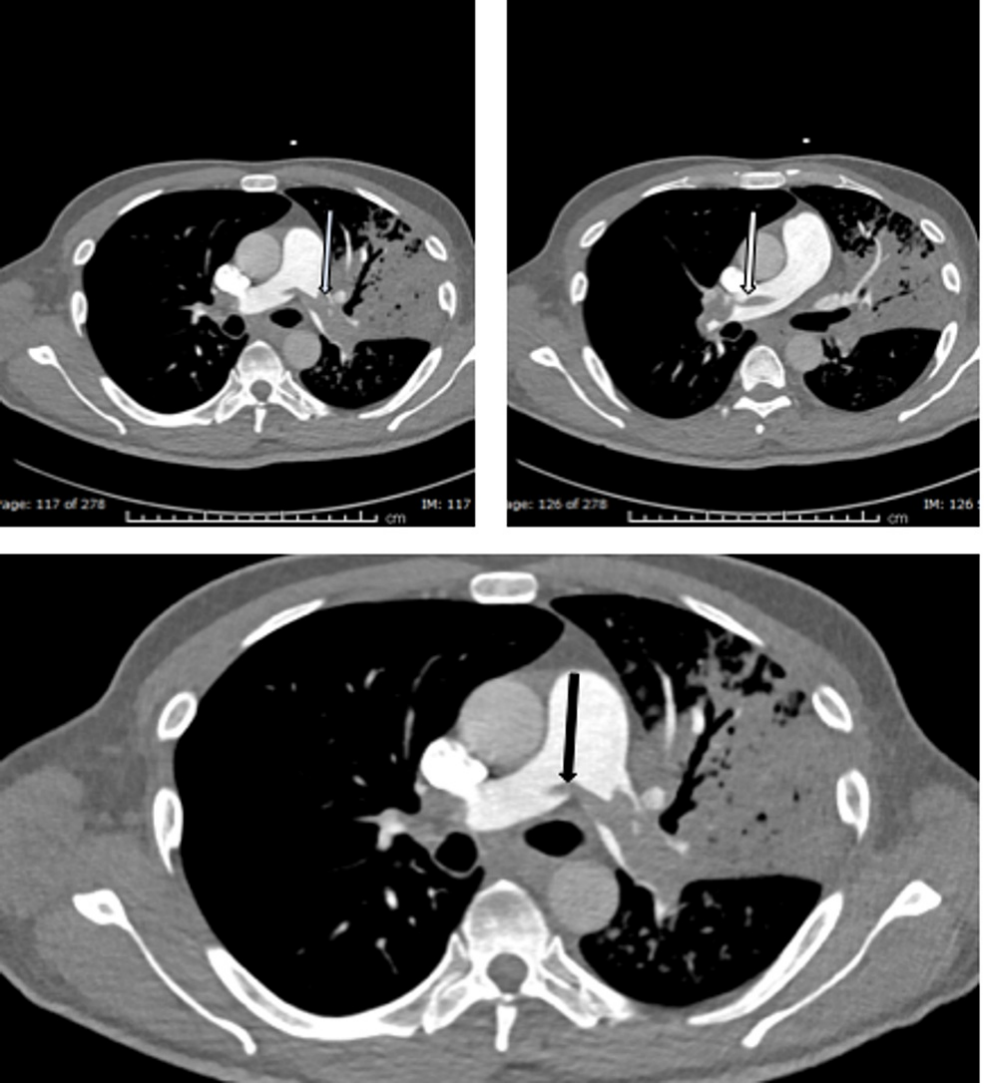
CT pulmonary angiogram (PA) demonstrating large filling defect within the main pulmonary artery (black arrow), which is extending to the right (white arrow) and left (blue arrow) pulmonary arteries representing saddle pulmonary embolism

Chest CT pulmonary angiogram (CTPA) showed saddle embolism extending to both pulmonary arteries and their major divisions (Figure 4). Echocardiogram showed normal global systolic LV function (ejection fraction (EF) 52%), moderate-severely dilated right ventricle (RV), and severely reduced RV function associated with septal motion suggestive of RV pressure overload. His right atrium is moderately dilated, and the pulmonary artery pressure is severely increased. The patient was moved to a high dependency unit and was given thrombolysis therapy (alteplase) as per the local protocol. He showed significant improvement in his clinical condition with improvement in his heart rate and shortness of breath.

He was started with anticoagulation as per the local protocol with enoxaparin and warfarin. The plan is to follow the local isolation protocol for PTB and optimize his international normalized ratio (INR) within the therapeutic range.

## Discussion

Our case is a clear example of pulmonary tuberculosis, which was complicated by unstable pulmonary embolism (PE) and deep vein thrombosis (DVT). He was treated with thrombolysis successfully. VTE can occur at the time of presentation of PTB or later in the course of the disease. Although VTE is very rare, the thrombogenic potential of tuberculosis has been reported in the literature before [3-4,6]. Other sites where VTE can occur in patients with TB are the hepatic veins [7] and cerebral venous sinuses [8].

Venous thromboembolism in patients with tuberculosis can be due to different causes. One of the most common causes is an inflammatory reaction that triggers elevated fibrinogen levels with impaired fibrinolysis, which is coupled with a decrease in the antithrombin III level and reactive thrombocytosis [9-10]. Secondly, patients with severe tuberculosis are often dehydrated. According to Poiseuille’s equation, when fluid in plasma is diminished due to dehydration, blood viscosity will increase and result in a reduced blood flow and increased prothrombotic state [9,11]. The third reason is attributed to the use of antitubercular drugs such as rifampicin. This drug is associated with a four times higher risk of deep vein thrombosis in a retrospective analysis of 1366 adult TB patients [9,12]. The occurrence of thrombosis usually happened two weeks from the commencement of antitubercular drugs [9]. Lastly, enlarged lymph nodes due to cell-mediated immunity can cause compressive effects on blood vessels, which result in vascular thrombosis [9,13-14].

Our case showed an unstable PE in a patient with a very clear diagnosis of PTB, which can be easily missed due to the nature of symptoms that are common to both conditions; however, unexplained tachycardia, mild leg swelling on careful examination, with raised a troponin level helped us investigate for VTE. Actually, even though there are many risk factors for PE, it is easy to miss it due to the poor specificity of the symptoms [15]. In acute PE, elevated heart rate is not only a diagnostic marker but also serves as one of the prognostic factors for mortality outcome, principally in-hospital mortality [16-17].

The patient was thrombolysed successfully with alteplase (50 mg) as per local protocol due to unstable PE as indicated by CTPA and echocardiogram findings. He tolerated it very well. Therefore, thrombolysis with alteplase should be considered in such patients with a low risk of bleeding or no contraindications. Another challenge in these patients is the drug interaction between ATT and anticoagulants like warfarin due to the effects of rifampicin on drug metabolism through cytochrome P450 [18]. Our patient was started on a therapeutic dose of enoxaparin (LMWH) and warfarin until the targeted INR level was achieved along with ATT. He will be referred to an anticoagulation clinic and infectious disease clinic after discharge.

## Conclusions

This case report highlights the increased risk of VTE in patients with pulmonary tuberculosis due to a hypercoagulable state associated with tuberculosis. This can lead to increased mortality, which is potentially preventable by prompt diagnosis and treatment. It also highlights the fact that thrombolysis is well-tolerated in patients with unstable PE with PTB who have no contraindications.

## Appendices

Data availability statement

The data that support the findings of this study are available from the corresponding author upon reasonable request.
